# The clinical value of fibrosis indices for predicting the hemorrhagic transformation in patients with acute ischemic stroke after intravenous thrombolysis

**DOI:** 10.3389/fnagi.2024.1492410

**Published:** 2024-11-25

**Authors:** Jiahao Chen, Xiaoqin Li, Rui Hu, Chuanchen Hu

**Affiliations:** ^1^Department of Neurology, Affiliated Jinhua Hospital, Zhejiang University School of Medicine, Jinhua, China; ^2^Department of Neurology, Yongkang First People’s Hospital, Jinhua, China

**Keywords:** acute ischemic stroke, hemorrhagic transformation, intravenous thrombolysis, non-alcoholic fatty liver disease, liver fibrosis

## Abstract

**Background:**

The incidence of stroke in China is approximately 343 per 100,000 people each year, the highest rate worldwide. Hemorrhagic transformation (HT), particularly symptomatic intracerebral hemorrhage (sICH) following acute ischemic stroke (AIS) with or without intravenous thrombolysis (IVT), can lead to rapid neurological deterioration, poor prognosis, and even death. Non-alcoholic fatty liver disease (NAFLD) has been identified as a risk factor for stroke occurrence and associated with poor long-term functional outcomes. Nonetheless, no studies have examined the association between liver fibrosis and HT in AIS patients who underwent IVT.

**Method:**

A total of 826 patients with AIS who underwent IVT were included in this study. We calculated nine validated liver fibrosis indices to assess the extent of liver fibrosis. HT was detected by follow-up cranial CT/MRI within 24 h post-IVT and was classified as either hemorrhagic infarction (HI) or parenchymal hematoma (PH). Symptomatic intracranial hemorrhage was defined as a sudden symptomatic neurological deterioration, indicated by an increase in (National Institutes of Health Stroke Scale) NIHSS score of 4 points or more.

**Result:**

The median values of fibrosis-4 (FIB-4), modified FIB-4 (mFIB-4), aspartate aminotransferase (AST)-platelet ratio index (APRI), Forns index, alanine aminotransferase (ALT)/AST (ARR), AST/ALT ratio-platelet ratio index (AARPRI), fibrosis quotient (FibroQ), and Fibrosis Index were significantly higher, while the fibrosis-5 (FIB-5) was significantly lower in the HT and sICH groups (all *P* < 0.001). After adjusting for potential confounders, all nine liver fibrosis indices remained associated with HT and sICH. Receiver operating characteristic (ROC) curve analysis revealed that the FibroQ score had the best predictive ability for HT (AUC = 0.707, CI = 0.652–0.762, *P* < 0.001), while FIB-4 had the best predictive ability for sICH (AUC = 0.802, CI = 0.711–0.892, *P* < 0.001).

**Conclusion:**

Liver fibrosis, as validated by FIB-4, mFIB-4, FIB-5, APRI, Forns index, ARR, AARPRI, FibroQ, and Fibrosis Index, was associated with HT and sICH in AIS patients after IVT.

## Introduction

In China, the incidence of stroke is approximately 343 per 100,000 people each year, the highest rate worldwide (Wang W. et al., 2017; GBD 2019 Stroke Collaborators, 2021). Patients with acute ischemic stroke (AIS) often experience early neurological deterioration during hospitalization and poor long-term functional outcomes, which adds to their financial burden and negatively impacts their quality of life (Helleberg et al., 2016). Currently, intravenous thrombolysis (IVT) and endovascular treatments, such as thrombectomy, are recommended for AIS treatment in the acute stage. Although IVT with recombinant tissue plasminogen activator (rt-PA) is effective in improving neurological prognosis, it also increases the risk of hemorrhagic transformation (HT) within 24 after admission (Strbian et al., 2011). HT, particularly symptomatic intracerebral hemorrhage (sICH), can lead to rapid neurological deterioration, poor prognosis, and even death (He et al., 2022).

Non-alcoholic fatty liver disease (NAFLD) encompasses a broad spectrum of liver illnesses, ranging from steatosis to non-alcoholic hepatitis, which can progress to liver fibrosis, cirrhosis, and hepatocellular carcinoma (Trauner et al., 2010). NAFLD and liver fibrosis have already been identified as risk factors for stroke occurrence (Kim et al., 2017; Parikh et al., 2019), particularly in patients with atrial fibrillation (AF) and are linked to worse functional outcomes in both the short term and long-term (Fandler-Höfler et al., 2021). Furthermore, liver fibrosis indices have been shown to be associated with hematoma expansion after primary intracerebral hemorrhage (ICH) (Wang et al., 2021), the prognosis of ICH (Parikh et al., 2020), and aneurysmal subarachnoid hemorrhage (Li et al., 2022). Interestingly, Yuan et al. (2020) found that fibrosis-4 (FIB-4), a representative index for measuring liver fibrosis, was associated with spontaneous hemorrhagic transformation (sHT) in AIS patients who did not receive any endovascular treatment. Moreover, this relationship remained significant after adjusting for confounders (odds ratio [OR] = 3.461, confidence interval [CI] = 1.404–8.531, *P* = 0.007). Nevertheless, they did not find a significant difference between subtypes of HT based on radiological characteristics. To date, no studies have specifically examined the association between liver fibrosis and HT (including sICH) in patients who underwent IVT. Herein, we hypothesize that liver fibrosis indices are associated with HT (including sICH) in patients with AIS after IVT, as well as with subtypes of HT.

## Materials and methods

### Patients

We conducted this retrospective study using data from the AIS database, which includes information from patients admitted to the Affiliated Jinhua Hospital of Zhejiang University School of Medicine. All patients included in the study underwent IVT between January 2019 and March 2024 in the emergency room before being admitted. The study included all consecutive patients over 18 years of age who received IVT with rt-PA at a dose of 0.9 mg/kg (maximum 90 mg) within 4.5 h of probable stroke onset. The ethics committees and institutional review boards of the Affiliated Jinhua Hospital of Zhejiang University School of Medicine approved the study and waived the requirement for informed consent due to the retrospective nature of the research and the anonymity of all data.

The diagnosis of AIS was confirmed through brain magnetic resonance imaging (MRI) or computed tomography (CT) after admission. The exclusion criteria were as follows: (1) unavailable data, (2) absence of follow-up CT or MRI within 24 h, (3) prior alcohol use (European Association for the Study of the Liver [EASL] et al., 2016), (4) presence of overt liver disease such as hepatitis, cirrhosis, or liver cancer, and (5) severe renal disease.

### Data collection

Baseline data were meticulously collected from the medical records of each patient, including admission demographic characteristics (age and sex) and medical history of AF, diabetes mellitus, hypertension, coronary heart disease (CHD), hyperlipidemia, and cigarette smoking. Prior antithrombotic (antiplatelet and anticoagulant) drugs usage and onset to treatment time (OTT) were also recorded from patients’ medical records. Stroke severity was assessed upon admission using the National Institutes of Health Stroke Scale (NIHSS) score (Brott et al., 1989). The modified Rankin Scale (mRS) score was used to assess the neurological function of each patient at admission. The Trial of ORG 10172 in Acute Stroke Treatment (TOAST) criteria were used to classify the subtypes of AIS (Adams et al., 1993). Fasting peripheral venous blood samples were collected within 24 h after admission to obtain laboratory data, including white blood cell count (WBC), glucose, aspartate aminotransferase (AST), alanine aminotransferase (ALT), gamma-glutamyl transpeptidase (GGT), total cholesterol (TC), albumin, alkaline phosphatase (AKP), platelet count (PLT), low-density lipoprotein cholesterol (LDL-C), high-density lipoprotein cholesterol (HDL-C), and international normalized ratio (INR).

### Calculation of liver fibrosis indices

**FIGURE 1:**
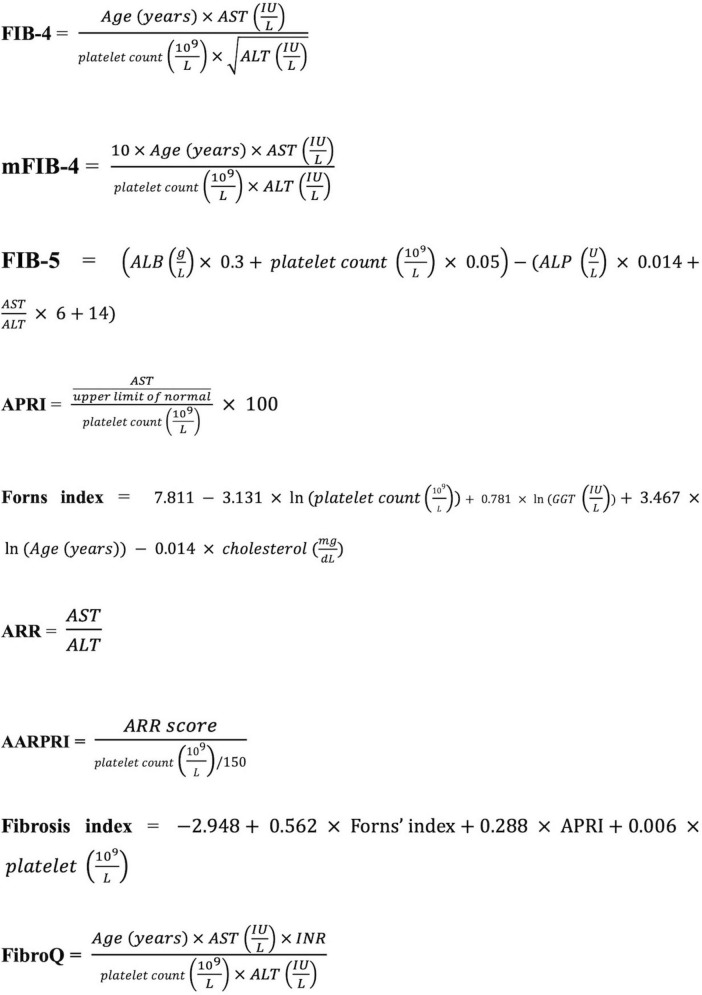
Formulas for liver fibrosis indices. AST, aspartate aminotransferase; ALT, alanine transaminase; ALB, albumin; ALP, alkaline phosphatase; GGT, gamma-glutamyl transpeptidase; INR: international normalized ratio; FIB-4, fibrosis-4; mFIB-4, modified FIB-4; FIB-5, fibrosis-5; APRI, AST/platelet ratio; ARR, ALT/AST ratio, AARPRI, AST/ALT ratio-platelet ratio; FibroQ, fibrosis quotient.

We selected nine fibrosis indices: fibrosis-4 (FIB-4), modified FIB-4 (mFIB-4), fibrosis-5 (FIB-5), AST/platelet ratio (APRI), Forns index, ALT/AST ratio (ARR), AST/ALT ratio-platelet ratio (AARPRI), fibrosis quotient (FibroQ), and the Fibrosis Index (Wai et al., 2003; Attallah et al., 2006; Sterling et al., 2006; Hsieh et al., 2009; Wang H. -W. et al., 2017; Crudele et al., 2023). All fibrosis indices were calculated using the formulas shown in Figure 1.

### Definition of HT and sICH

Patients with suspected AIS underwent brain CT examination prior to IVT, and subsequent cranial CT or MRI was performed within 24 h post-IVT to screen for HT. In cases of clinical symptom deterioration, immediate imaging (CT/MRI) was conducted. HT was radiologically classified into hemorrhagic infarction (HI) and parenchymal hematoma (PH) (Hacke et al., 1995). HT was defined as sICH if the patient experienced symptomatic neurological deterioration, indicated by an increase in the NIHSS score of 4 points or more.

### Statistical analyses

Data distribution normality was assessed using the Kolmogorov–Smirnov test. Continuous variables with normal distributions were expressed as mean ± standard deviation, while those with non-normal distributions were expressed as median with interquartile range. Categorical variables were expressed as relative frequency and percentage. Continuous variables were compared using the Student’s *t*-test or the Mann–Whitney U test, as appropriate. Categorical variables were compared using the chi-square test or Fisher’s exact test. Statistical comparisons of fibrosis indices among different subtypes of HT were performed using one-way analysis of variance (ANOVA) or the Kruskal–Wallis test. After adjusting for conventional confounding factors and significant variables (*P* < 0.1) identified in univariate logistic regression analysis (for HT occurrence: age, gender, AF, hyperlipemia, baseline NIHSS and WBC; for sICH: age, gender, AF, SBP, TOAST classification, baseline NIHSS, WBC and glucose), a multivariate binary logistic regression was conducted to determine whether the fibrosis indices were independent predictors of HT and sICH after AIS. The predictive capacity of each fibrosis index for identifying possible HT and sICH was assessed using receiver operating characteristic (ROC) curves. A two-tailed *P* < 0.05 was considered statistically significant. All statistical analyses were performed using R version 4.1.2.

## Results

**FIGURE 2:**
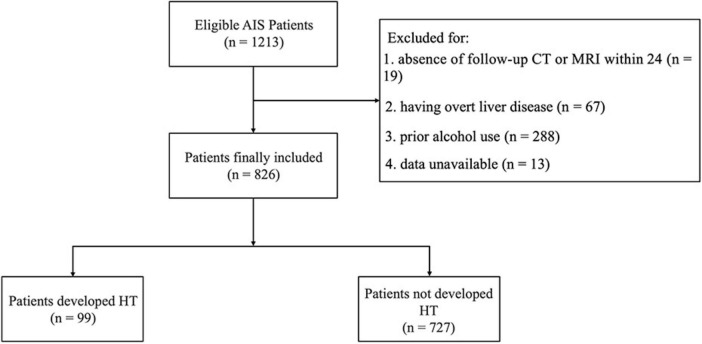
Flow diagram of patient enrollment. AIS, acute ischemic stroke; HT, hemorrhagic transformation.

A total of 826 AIS patients who underwent IVT were included in this study, with a median age of 70 years (range 59–78). Of these patients, 537 (65.0%) were male. Among the cohort, 99 patients (11.9%) developed HT, while 727 patients (88.0%) did not (Figure 2). Besides, 23 (2.8%) patients developed sICH.

### Baseline characteristics of patients with and without HT

The baseline characteristics and laboratory findings of patients with AIS are detailed in Table 1. The HT group had significantly higher age, NIHSS score at admission, mRS score at discharge, hospitalization days, WBC count levels, AST levels (all *P* < 0.001), and lower PLT levels (*P* = 0.002). In addition, patients in the HT group exhibited higher FIB-4, mFIB-4, APRI, Forns index, ARR, AARPRI, FibroQ, and Fibrosis Index, and a lower FIB-5 score (all *P* < 0.001).

**TABLE 1:** Demographics and clinical characteristics between patients with or without HT.

Variables	Non-HT (*n* = 727)	HT (*n* = 99)	*P*-value
**Demographics**			
Age, years (IQR)	68.0 (58.0–76.0)	77.0 (71.0–84.0)	**< 0.001**
Gender (male, %)	484 (66.6%)	53 (53.5%)	**0.015**
**Vascular risk factor**			
Hypertension, *n* (%)	580 (79.8%)	83 (83.8%)	0.414
Diabetes mellitus, *n* (%)	166 (22.8%)	22 (22.2%)	0.993
Atrial fibrillation, *n* (%)	118 (16.2%)	43 (43.4%)	**< 0.001**
CHD, *n* (%)	47 (6.5%)	6 (6.1%)	1.000
Hyperlipidemia, *n* (%)	134 (18.4%)	11 (11.1%)	0.098
History of smoking, *n* (%)	246 (33.8%)	31 (31.3%)	0.670
History of stroke, *n* (%)	112 (15.4%)	13 (13.1%)	0.658
**Clinical information**			
SBP at admission, mmHg (IQR)	151.0 (136.8–167.0)	158.0 (139.0–169.5)	0.133
DBP at admission, mmHg (IQR)	85.0 (76.0–95.0)	87.0 (74.5–97.0)	0.866
DNT, min (IQR)	37.0 (29.0–50.0)	38.0 (31.8–50.3)	0.398
NIHSS at admission (IQR)	3.0 (1.0–5.0)	5.0 (2.0–10.5)	**< 0.001**
mRS at discharge (IQR)	2.0 (1.0–3.0)	3.5 (2.0–5.0)	**< 0.001**
Hospitalization, days (IQR)	8.0 (7.0–9.0)	12.0 (8.0–17.0)	**< 0.001**
OTT, hours (IQR)	2.5 (1.8–3.5)	2.6 (1.8–3.6)	0.534
Prior antiplatelet/anticoagulant use	161 (22.1%)	20 (20.2%)	0.757
**TOAST classification**			**< 0.001**
Large artery atherosclerosis, *n* (%)	238 (32.7%)	28 (28.3%)	
Cardioembolism, *n* (%)	127 (17.5%)	48 (48.5%)	
Small vessel occlusion, *n* (%)	285 (39.2%)	12 (12.1%)	
Other determined, *n* (%)	7 (1.0%)	0 (0.0%)	
Undetermined, *n* (%)	70 (9.6%)	9 (11.1%)	
**Laboratory signs**			
WBC, 10^9^/L (IQR)	6.8 (5.6–8.4)	7.8 (6.5–9.9)	**< 0.001**
Glucose, mmol/L (IQR)	5.1 (4.6–6.1)	5.4 (4.6–6.6)	0.147
AST, U/L (IQR)	20.8 (17.7–25.3)	24.7 (20.4–30.5)	**< 0.001**
ALT, U/L (IQR)	15.8 (11.7–21.3)	14.5 (12.0–18.5)	0.235
GGT, U/L (IQR)	24.6 (17.1–42.7)	24.4 (16.7–38.8)	0.520
TC, mmol/L (IQR)	1.7 (1.1–3.6)	2.3 (1.0–4.2)	0.290
LDL-C, mmol/L (IQR)	2.9 (2.3–3.4)	2.6 (2.1–3.1)	**0.002**
HDL-C, mmol/L (IQR)	1.2 (1.0–1.4)	1.3 (1.0–1.5)	**0.019**
Albumin, g/L (IQR)	38.2 (36.1–40.3)	37.7 (35.1–40.6)	0.325
AKP, IU/L (IQR)	79.0 (65.0–95.0)	82.0 (70.0–95.5)	0.200
Platelet, 10^9^/L (IQR)	200.0 (158.0–240.5)	184.0 (137.0–229.5)	**0.002**
INR	1.03 (0.98–1.08)	1.05 (1.02–1.13)	**< 0.001**
**Fibrosis indices**			
FIB-4 (IQR)	1.8 (1.2–2.7)	2.8 (1.7–4.1)	**< 0.001**
mFIB-4 (IQR)	4.5 (2.8–4.5)	7.1 (4.6–10.7)	**< 0.001**
FIB-5 (IQR)	−1.7 (−5.6, −1.8)	−4.7 (−8.9, −1.1)	**< 0.001**
APRI (IQR)	0.3 (0.2–0.4)	0.4 (0.3–0.5)	**< 0.001**
FORNS (IQR)	8.3 (7.4–9.3)	9.1 (6.4–8.7)	**< 0.001**
ARR (IQR)	1.3 (1.0–1.7)	1.7 (1.4–2.0)	**< 0.001**
AARPRI (IQR)	1.0 (0.7–1.5)	1.4 (1.0–2.1)	**< 0.001**
FibroQ (IQR)	4.7 (2.9–7.2)	8.4 (4.9–12.2)	**< 0.001**
Fibrosis index (IQR)	3.1 (2.7–3.4)	3.4 (3.0–3.8)	**< 0.001**

HT, hemorrhagic transformation; NIHSS, National Institutes of Health Stroke Scale; CHD, coronary heart disease; SBP, systolic blood pressure; DBP, diastolic blood pressure; mRS, modified Rankin Scale; DNT, door to Needle Time; TOAST, trial of ORG 10172 in acute stroke treatment; OTT, onset to treatment time; WBC, white blood cell; AST, aspartate aminotransferase; ALT, alanine aminotransferase; GGT, gamma-glutamyl transpeptidase; TC, total cholesterol; AKP, alkaline phosphatase; PLT, platelet count; LDL-C, low-density lipoprotein cholesterol; HDL-C, High-density lipoprotein cholesterol; INR, international normalized ratio. *P*-value < 0.05 was marked as bold.

### Baseline characteristics of patients with and without sICH

Table 2 shows the comparison between patients with sICH and those without sICH. The sICH group had a significantly older age (*P* = 0.002), a higher proportion of AF (*P* < 0.001), and significantly higher SBP at admission (*P* = 0.037), NIHSS score at admission (*P* < 0.001), mRS score at discharge (*P* < 0.001), hospitalization days (*P* < 0.001), WBC count (*P* < 0.001), glucose level (*P* = 0.008), and AST level (*P* = 0.001). Moreover, the sICH group had higher FIB-4, mFIB-4, APRI, Forns index, ARR, AARPRI, FibroQ, and Fibrosis Index, as well as lower PLT count, and FIB-5 score (all *P* < 0.001).

**TABLE 2:** Demographics and clinical characteristics between patients with or without sICH.

Variables	Non-sICH (*n* = 803)	sICH (*n* = 23)	*P*-value
**Demographics**			
Age, years (IQR)	69.0 (59.0–77.5)	78.0 (71.0–85.5)	**0.002**
Gender (male,%)	524 (65.3%)	13 (56.5%)	0.520
**Vascular risk factor**			
Hypertension, *n* (%)	642 (80.0%)	21 (91.3%)	0.279
Diabetes mellitus, *n* (%)	183 (22.8%)	5 (21.7%)	1.000
Atrial fibrillation, *n* (%)	151 (18.8%)	10 (43.5%)	**< 0.001**
CHD, *n* (%)	53 (6.6%)	0 (0.0%)	0.400
Hyperlipidemia, *n* (%)	143 (17.8%)	2 (8.7%)	0.393
History of smoking, *n* (%)	269 (33.5%)	8 (34.8%)	1.000
History of stroke, *n* (%)	120 (14.9%)	5 (21.7%)	0.548
**Clinical information**			
SBP at admission, mmHg (IQR)	152.0 (136.0–167.0)	166.0 (143.0–181.5)	**0.037**
DBP at admission, mmHg (IQR)	85.0 (76.0–95.0)	90.0 (77.0–97.5)	0.390
DNT, min (IQR)	37.5 (29.0–50.0)	38.5 (32.0–46.5)	0.685
NIHSS at admission (IQR)	3.0 (1.0–6.0)	10.0 (4.0–13.5)	**< 0.001**
mRS at discharge (IQR)	2.0 (1.0–3.0)	4.5 (4.0–5.0)	**< 0.001**
Hospitalization days (IQR)	8.0 (7.0–10.0)	17 (10.5–23.0)	**< 0.001**
OTT, hours (IQR)	2.6 (1.8–3.6)	2.8 (2.2–4.1)	0.022
**TOAST classification**			**0.005**
Large artery atherosclerosis, *n* (%)	255 (31.8%)	11 (47.8%)	
Cardioembolism, *n* (%)	165 (20.5%)	10 (43.5%)	
Small vessel occlusion, *n* (%)	296 (36.9%)	1 (4.3%)	
Other determined, *n* (%)	7 (0.8%)	0 (0.0%)	
Undetermined, *n* (%)	80 (10.0%)	1 (4.3%)	
**Laboratory signs**			
WBC, 10^9^/L (IQR)	6.9 (5.6–8.5)	9.1 (7.4–11.0)	**< 0.001**
Glucose, mmol/L (IQR)	5.1 (4.6–6.1)	6.6 (5.2–7.3)	**0.008**
AST, U/L (IQR)	21.1 (17.9–25.8)	27.0 (20.6–33.6)	**0.001**
ALT, U/L (IQR)	15.7 (11.7–21.0)	14.2 (12.1–16.9)	0.339
GGT, U/L (IQR)	24.5 (17.1–42.5)	27.2 (15.5–36.5)	0.526
TC, mmol/L (IQR)	1.7 (1.11–3.7)	3.2 (0.9–3.9)	0.985
LDL-C, mmol/L (IQR)	2.8 (2.3–3.4)	2.1 (1.9–2.9)	**0.003**
HDL-C, mmol/L (IQR)	1.2 (1.0–1.4)	1.4 (1.2–1.6)	**< 0.001**
Albumin, g/L (IQR)	38.2 (36.0–40.3)	37.6 (36.1–40.5)	0.887
AKP, IU/L (IQR)	79.0 (66.0–95.0)	82.0 (73.0–87.0)	0.283
PTA, % (IQR)	94.0 (87.0–101.0)	92.0 (77.0–99.8)	0.196
Platelet, 10^9^/L (IQR)	200.0 (157.0–240.0)	121.0 (121.0–185.0)	**< 0.001**
**Fibrosis scores**			
FIB-4 (IQR)	1.9 (1.3–2.8)	3.7 (2.4–5.4)	**< 0.001**
mFIB-4 (IQR)	4.7 (2.9–7.3)	9.8 (5.3–14.3)	**< 0.001**
FIB-5 (IQR)	−2.0 (−5.8 to 1.6)	−7.6 (−10.1 to −3.9)	**< 0.001**
APRI (IQR)	0.3 (0.2–0.4)	0.5 (0.4–0.6)	**< 0.001**
FORNS (IQR)	8.4 (7.4–9.3)	10.0 (8.8–10.4)	**< 0.001**
ARR (IQR)	0.3 (0.2–0.4)	0.5 (0.4–0.6)	**< 0.001**
AARPRI (IQR)	1.0 (0.7–1.5)	1.9 (1.2–2.5)	**< 0.001**
FibroQ (IQR)	4.8 (3.0–7.7)	12.3 (5.6–14.9)	**< 0.001**
Fibrosis index (IQR)	3.1 (2.7–3.4)	3.6 (3.3–3.8)	**< 0.001**

sICH, symptomatic intracranial hemorrhage; NIHSS, National Institutes of Health Stroke Scale; CHD, coronary heart disease; SBP, systolic blood pressure; DBP, diastolic blood pressure; mRS, modified Rankin Scale; DNT, door to Needle Time; TOAST, Trial of ORG 10172 in Acute Stroke Treatment; OTT, onset to treatment time; WBC, white blood cell; AST, aspartate aminotransferase; ALT, alanine aminotransferase; GGT, gamma-glutamyl transpeptidase; TC, total cholesterol; AKP, alkaline phosphatase; PLT, platelet count; LDL-C, low-density lipoprotein cholesterol; HDL-C, High-density lipoprotein cholesterol; INR, international normalized ratio. *P*-value < 0.05 was marked as bold.

### Association between fibrosis indices and HT (and sICH) following IVT

**FIGURE 3:**
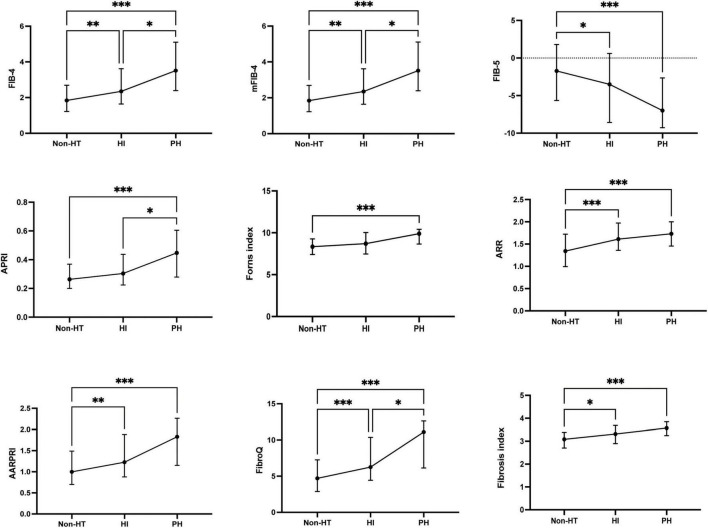
The fibrosis indices in the subcategorized groups of HT. Each data point and error bar correspond to the median and interquartile range of the fibrosis indices by the subcategorized groups of HT. HT, hemorrhagic transformation; HI, hemorrhagic infarct; PH, parenchymal hematoma. *P < 0.01; **P < 0.01; ***P < 0.001.

**FIGURE 4:**
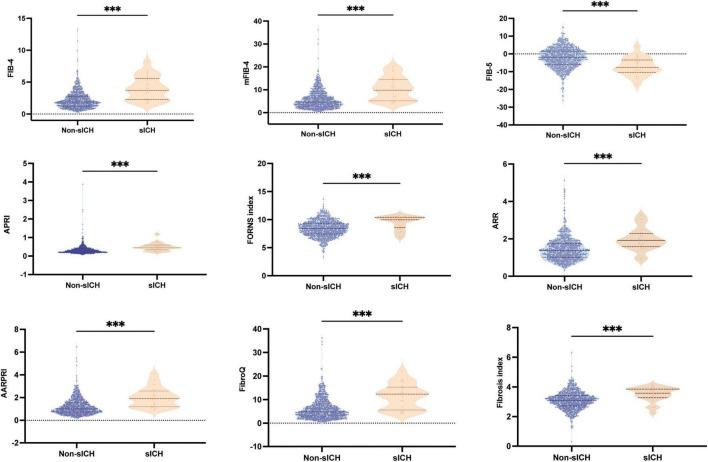
Boxplots of the fibrosis indices showing the distribution in the sICH group and Non-sICH group. sICH, symptomatic intracranial hemorrhage. ***P < 0.001.

Baseline fibrosis indices were significantly different between patients with and without HT and sICH. Based on the characteristics of HT observed in CT/MRI imaging, HT was subdivided into HI and PH. As shown in Figure 3, there were significant differences in fibrosis indices between patients with HI or PH and those without HT. For PH versus Non-HT, all nine fibrosis indices were showed significant differences (*P* < 0.001) and there were statistically significant differences in seven fibrosis indices for differentiating HI and Non-HT (FIB-4: *P* < 0.01, mFIB-4: *P* < 0.01, FIB-5: *P* < 0.05, ARR: *P* < 0.001, AARPRI: *P* < 0.01, FibroQ: *P* < 0.001, Fibrosis index: *P* < 0.05). Furthermore, several indices, including FIB-4 (*P* < 0.05), mFIB-4 (*P* < 0.05), APRI (*P* < 0.05), and FibroQ scores (*P* < 0.05), demonstrated significant differences between patients with HI and those with PH. As for sICH, there were significant differences in all nine fibrosis indices between patients with sICH and those without, as shown in Figure 4 (all *P* < 0.001).

In univariate binary logistic regression analyses, higher FIB-4, mFIB-4, APRI, Forns index, ARR, AARPRI, FibroQ, and Fibrosis Index, as well as a lower FIB-5 score, were associated with an increased risk of HT and sICH (*P* < 0.001), as shown in the crude model (Table 3). After adjusting for relevant potential confounding factors identified in the overall univariate analysis (Supplementary Table 1), higher FIB-4 (Odds ratio (OR) = 1.340, confidence interval (CI) = 1.170–1.544, *P* < 0.001; OR = 1.358, CI = 1.184–1.569, *P* < 0.001), mFIB-4 (OR = 1.803, CI = 1.031–1.139, *P* = 0.008; OR = 1.090, CI = 1.037–1.147, *P* = 0.004), APRI (OR = 2.370, CI = 1.268–4.466, *P* = 0.020; OR = 2.388, CI = 1.285–4.516, *P* = 0.019), Forns index (OR = 1.270, CI = 1.056–1.524, *P* = 0.032; OR = 1.280, CI = 1.063–1.538, *P* = 0.028), AARPRI (OR = 1.512, CI = 1.171–1.949, *P* = 0.007; OR = 1.567, CI = 1.211–2.025, *P* = 0.004), FibroQ (OR = 1.081, CI = 0.918–0.991, *P* = 0.008; OR = 1.088, CI = 1.039–1.141, *P* = 0.003) and lower FIB-5 (OR = 0.953, CI = 1.031–1.139, *P* = 0.008; OR = 0.948, CI = 0.912–0.986, *P* = 0.023) scores were still associated with an increased risk of HT and sICH, respectively.

**TABLE 3:** Multivariate logistic regression of the association between liver fibrosis indices and HT.

	Crude model		Model 2		Model 3	
**Variables**	**OR (95% CI)**	***P*-value**	**Adjusted OR (95% CI)**	***P*-value**	**Adjusted OR (95% CI)**	***P*-value**
**For HT occurrence**						
FIB-4	1.585 (1.414–1.785)	**< 0.001**	1.398 (1.231–1.598)	**< 0.001**	1.340 (1.170–1.544)	**< 0.001**
mFIB-4	1.151 (1.107–1.199)	**< 0.001**	1.091 (1.042–1.144)	**0.002**	1.083 (1.031–1.139)	**0.008**
FIB-5	0.914 (0.886–0.943)	**< 0.001**	0.953 (0.919–0.988)	**0.029**	0.953 (0.918–0.991)	**0.039**
APRI	3.423 (1.867–6.706)	**< 0.001**	2.801 (1.559–5.246)	**0.004**	2.370 (1.268–4.466)	**0.020**
FORNS	1.243 (1.118–1.385)	**< 0.001**	1.240 (1.023–1.066)	**0.034**	1.270 (1.056–1.524)	**0.032**
ARR	1.936 (1.520–2.467)	**< 0.001**	1.394 (1.049–1.833)	**0.049**	1.250 (0.910–1.686)	0.232
AARPRI	2.007 (1.621–2.500)	**< 0.001**	1.564 (1.233–1.990)	**0.002**	1.512 (1.171–1.949)	**0.007**
FibroQ	1.146 (1.106–1.190)	**< 0.001**	1.096 (1.050–1.146)	**< 0.001**	1.081 (1.032–1.132)	**0.006**
Fibrosis index	2.631 (1.922–3.638)	**< 0.001**	1.730 (1.119–2.627)	**0.034**	1.529 (0.935–2.428)	0.142
**For sICH occurrence**						
FIB-4	1.590 (1.355–1.880)	**< 0.001**	1.494 (1.252–1.786)	**< 0.001**	1.358 (1.184–1.569)	**< 0.001**
mFIB-4	1.167 (1.103–1.237)	**< 0.001**	1.136 (1.062–1.216)	**0.002**	1.090 (1.037–1.147)	**0.004**
FIB-5	0.875 (0.827–0.925)	**< 0.001**	0.899 (0.844–0.959)	**0.006**	0.948 (0.912–0.986)	**0.023**
APRI	2.985 (1.379–6.183)	**0.011**	3.006 (1.310–6.230)	**0.013**	2.388 (1.285–4.516)	**0.019**
FORNS	1.726 (1.332–2.254)	**< 0.001**	1.582 (1.144–2.172)	**0.018**	1.280 (1.063–1.538)	**0.028**
ARR	2.262 (1.511–3.276)	**< 0.001**	1.829 (1.136–2.781)	**0.025**	1.301 (0.946–1.758)	0.161
AARPRI	2.367 (1.711–3.275)	**< 0.001**	2.034 (1.420–2.900)	**< 0.001**	1.567 (1.211–2.025)	**0.004**
FibroQ	1.157 (1.098–1.221)	**< 0.001**	1.131 (1.061–1.206)	**0.001**	1.088 (1.039–1.141)	**0.003**
Fibrosis index	2.916 (1.653–5.116)	**0.002**	2.297 (1.028–4.572)	0.063	1.547 (0.943–2.461)	0.133

HT, hemorrhagic transformation; sICH, symptomatic intracranial hemorrhage. For HT occurrence: Crude model did not adjust any confounders Model 2 adjusted for age and gender Model 3 adjusted for covariates from Model 2 and further adjusted atrial fibrillation (AF), hyperlipemia, baseline NIHSS and WBC. For sICH occurrence: Crude model did not adjust any confounders Model 2 adjusted for age and gender Model 3 adjusted for covariates from Model 2 and further adjusted atrial fibrillation (AF), systolic blood pressure (SBP), TOAST classification, baseline NIHSS, WBC and glucose. *P*-value < 0.05 was marked as bold.

**FIGURE 5:**
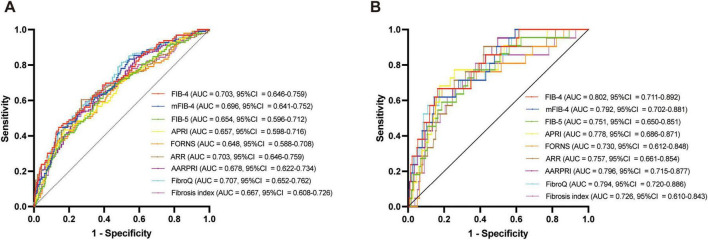
ROC curves of the fibrosis indices for predicting HT and sICH. **(A)** HT; **(B)** sICH.

ROC analysis was conducted to evaluate the predictive ability of fibrosis indices for HT and sICH in patients with AIS who underwent IVT (Figure 5). Among all fibrosis indices, the FibroQ score (AUC = 0.707, 95% CI = 0.652–0.762, *P* < 0.001), FIB-4 score (AUC = 0.703, 95% CI = 0.646–0.759, *P* < 0.001) and mFIB-4 score (AUC = 0.696, 95% CI = 0.641–0.752, *P* < 0.001) were top three predictive indicators according to there AUCs. And for sICH, the FIB-4 score (AUC = 0.802, 95% CI = 0.711–0.892, *P* < 0.001), AARPRI score (AUC = 0.796, 95% CI = 0.715–0.877, *P* < 0.001) and FibroQ score (AUC = 0.794, 95% CI = 0.702–0.886, *P* < 0.001) had the top three predictive ability for sICH. Additional details of the fibrosis indices, including cutoff values, sensitivity, and specificity, are presented in Table 4.

**TABLE 4:** The ability of liver fibrosis indices in predicting HT and sICH.

Variables	AUC (95% CI)	*P*-value	Cutoff value	Sensitivity (%)	Specificity (%)
**For HT occurrence**					
FIB-4	0.703 (0.646–0.759)	**< 0.001**	3.293	85.9	44.8
mFIB-4	0.696 (0.641–0.752)	**< 0.001**	9.171	85.9	44.8
FIB-5	0.654 (0.596–0.712)	**< 0.001**	−3.090	60.1	64.3
APRI	0.657 (0.598–0.716)	**< 0.001**	0.413	82.7	43.3
FORNS	0.648 (0.588–0.708)	**< 0.001**	9.845	85.9	70.8
ARR	0.676 (0.624–0.728)	**< 0.001**	1.452	60.4	72.9
AARPRI	0.678 (0.622–0.734)	**< 0.001**	1.013	51.6	76.0
FibroQ	0.707 (0.652–0.762)	**< 0.001**	3.316	60.8	72.6
Fibrosis index	0.667 (0.608–0.726)	**< 0.001**	3.316	70.6	59.4
**For sICH occurrence**					
FIB-4	0.802 (0.711–0.892)	**< 0.001**	3.293	83.6	66.7
mFIB-4	0.792 (0.702–0.881)	**< 0.001**	9.601	85.6	61.9
FIB-5	0.751 (0.650–0.851)	**< 0.001**	−3.798	63.5	77.3
APRI	0.778 (0.686–0.871)	**< 0.001**	0.372	74.3	77.3
FORNS	0.730 (0.612–0.848)	**< 0.001**	9.643	81.2	66.7
ARR	0.757 (0.661–0.854)	**< 0.001**	1.456	58.0	90.5
AARPRI	0.796 (0.715–0.877)	**< 0.001**	1.827	84.3	61.9
FibroQ	0.794 (0.702–0.886)	**< 0.001**	9.426	82.8	66.7
Fibrosis index	0.726 (0.610–0.843)	**< 0.001**	3.244	62.6	81.0

HT, hemorrhagic transformation; sICH, symptomatic intracranial hemorrhage; AUC, area under curve. *P*-value < 0.05 was marked as bold.

## Discussion

In the present study, we explored the association between fibrosis indices and the risk of HT in patients who received IVT for AIS. We evaluated nine readily available fibrosis indices, which were independently associated with the risk of HT. Furthermore, ROC analysis demonstrated that these fibrosis indices have excellent predictive value for HT (including sICH).

Previous studies have established that liver diseases, including cirrhosis and NAFLD, are risk factors for cardiovascular and cerebrovascular diseases across the general population (Lawlor et al., 2014; Ekstedt et al., 2015; Targher et al., 2016). Recent research has increasingly focused on the relationship between liver fibrosis indices and stroke occurrence and functional outcomes. For example, two recent studies demonstrated that liver fibrosis, as validated by transient elastography (TE), predicts all-cause and cardiovascular mortality during follow-up in patients with AIS or transient ischemic attack (TIA) (Kim et al., 2017; Baik et al., 2019). Moreover, several studies have shown that FIB-4 is associated with sICH, functional outcomes, and mortality in AIS patients who received IVT (Norata et al., 2023; Toh et al., 2023). Notably, Yuan et al. (2020) found that liver fibrosis, measured using FIB-4, was an independent predictor of HT in patients who did not receive any revascularization therapy. However, they did not detect significant differences between HT subtypes according to the ECASS classification. Consistent with these findings, our study highlights a stronger association between liver fibrosis indices and HT in AIS patients who underwent IVT.

Nevertheless, the underlying mechanisms linking liver fibrosis with the risk of HT and sICH in patients with AIS who underwent IVT remain unclear and warrant further investigation. Several potential mechanisms may explain this association. Firstly, liver fibrosis is known to be associated with vascular endothelial dysfunction. Steatotic hepatocytes release more extracellular vesicles than normal hepatocytes, which can significantly alter microRNA expression profiles. Of note, microRNA-1 (miR-1) has been recently found to be highly expressed in vascular endothelial cells (VEC). However, its physiological role in cardiovascular and cerebrovascular diseases has not been fully elucidated. Jiang et al. (2020) demonstrated that co-culturing VECs with steatotic hepatocytes resulted in increased expression of proinflammatory molecules in VECs, enhanced NF-κB activity, and reduced Kruppel-like factor 4 expression. Furthermore, cerebral vascular endothelial dysfunction, which is associated with blood-brain barrier (BBB) disruption, has been shown to be a significant factor in HT following AIS (Yang et al., 2019). Secondly, liver fibrosis can lead to a hypercoagulable state by disrupting the expression of prothrombotic factors (Assy et al., 2005; Kotronen et al., 2011; Verrijken et al., 2014). Interestingly, Ryu et al. (2023) found that hypercoagulability, as assessed by thrombo-elastography parameters, may negatively predict the risk of HT and functional outcomes in AIS patients. Thirdly, systemic inflammation likely plays a critical role in the progression from liver fibrosis to HT in AIS patients who have undergone IVT. Several studies have explored the relationship between NAFLD and systemic inflammation. For instance, du Plessis et al. (2015) found that as the histological severity of NAFLD increases, levels of inflammatory factors such as interleukin-8 (IL-8), tumor necrosis factor-alpha (TNF-α), and chemokine (C-C motif) ligand 3 (CCL-3) also rise. Another study (Wagner et al., 2023) reported that matrix metalloproteinase-9 (MMP-9) levels were significantly higher in non-alcoholic steatohepatitis (NASH) patients compared to controls (12.6 ± 6.5 vs. 8.1 ± 3.5, *P* < 0.01). Fourthly, liver fibrosis has been associated with lipidosis, which may contribute to increased carotid intimal-medial thickness and carotid plaques (Weng et al., 2018; Beer et al., 2023).

In this retrospective study, we investigated the predictive abilities of easily accessible liver fibrosis indices for the occurrence of HT, including sICH. We observed that eight indices (FIB-4, mFIB-4, APRI, Forns index, ARR, AARPRI, FibroQ, and Fibrosis Index) were elevated, while the FIB-5 score was significantly decreased in patients with HT, including sICH. FIB-4, mFIB-4, and FibroQ scores exhibited significant differences between HI versus non-HT and PH versus HI. This suggests that these indices can predict HT occurrence with varying severity. Among the nine indices, FibroQ demonstrated the best predictive ability for HT, while FIB-4 was the most effective for sICH. Both FibroQ and FIB-4 include elements such as age, AST, PLT, and ALT, which have been identified as risk factors for unfavorable outcomes in cerebrovascular diseases (Wang et al., 2021, 2022; Li et al., 2022; Eto et al., 2024), aligning with our results. Additionally, FIB-4 and FibroQ showed significant differences between HI and PH, indicating that some liver fibrosis indices, particularly FIB-4, have better discriminative ability for HT and its subtypes in AIS patients who underwent IVT. This may be due to liver dysfunction affecting the clearance of rt-PA, leading to increased plasma tPA levels and potentially higher HT rates (Leebeek and Rijken, 2015). Furthermore, studies have reported that low LDL levels can also reduce rt-PA clearance (Okabe et al., 1992), and our study found that FIB-4 and FibroQ were negatively associated with LDL levels (Supplementary Figure 1).

Our study has several limitations. First, due to its retrospective nature, we cannot establish a causal relationship between liver fibrosis indices and HT. Second, being a single-center study, there may be patient selection bias affecting the results. Consequently, further prospective multicenter studies are needed to validate our findings. Third, while we used nine simple, non-invasive, and validated indices to assess liver fibrosis, we lacked liver elastography or biopsy data to confirm the exact presence of liver fibrosis in our study population. Fourth, we excluded AIS patients who reported alcohol consumption, which may have reduced our sample size, as a significant proportion of patients might not accurately recall their alcohol intakes.

## Conclusion

In conclusion, our study demonstrated that nine liver fibrosis indices are associated with an increased risk of HT, including sICH. Among these indices, the FibroQ score exhibited the best predictive ability for HT, while the FIB-4 score was most effective for sICH.

## Data Availability

The original contributions presented in this study are included in this article/Supplementary material, further inquiries can be directed to the corresponding author.

## Supplementary material

Supplementary Figure 1 The spearman’s rank correlation between two fibrosis indices and LDL- C. LDL-C, low-density lipoprotein cholesterol.
