# Supplementary material for: The clinical value of fibrosis indices for predicting the hemorrhagic transformation in patients with acute ischemic stroke after intravenous thrombolysis
Source: Front Aging Neurosci. 2024 Nov 25;16:1492410. doi: 10.3389/fnagi.2024.1492410 (PMC11625795; doi:10.3389/fnagi.2024.1492410)
Supplement: Supplementary file 1 [file Table_1.docx]

Supplementary table1: Univariate analysis of the association between variables and HT.

| Variables | **HT occurrence** | | **sICH occurrence** | |
| --- | --- | --- | --- | --- |
|  | OR (95%CI) | P-value | OR (95%CI) | P-value |
| **Demographics** | | | | |
| Age | 1.057 (1.040-1.075) | **<0.001** | 1.059 (1.026-1.096) | **0.004** |
| Gender (female) | 1.729 (1.209-2.466) | **0.011** | 1.445 (0.704-2.906) | 0.389 |
| **Vascular risk factor** | | | | |
| Hypertension | 1.315 (0.833-2.160) | 0.342 | 2.633 (0.909-11.574) | 0.194 |
| Diabetes mellitus | 0.966 (0.623-1.458) | 0.892 | 0.941 (0.375-2.077) | 0.906 |
| Atrial ﬁbrillation | 3.963 (2.726-5.746) | **<0.001** | 3.321 (1.6106.717) | **<0.001** |
| CHD | 0.933 (0.416-1.848) | 0.878 | - | |
| Hyperlipidemia | 0.553 (0.308-0.930) | **0.076** | 0.440 (0.999-1.275) | 0.270 |
| History of smoking | 0.891 (0.605-1.294) | 0.618 | 1.059 (0.493-2.159) | 0.898 |
| History of stroke | 0.830 (0.480-1.361) | 0.554 | 1.581 (0.627-3.506) | 0.374 |
| **Clinical Information** | | | | |
| SBP at admission | 1.007 (0.999-1.104) | 0.172 | 1.020 (1.005-1.035) | **0.028** |
| DBP at admission | 1.000 (0.989-1.013) | 0.891 | 1.010 (0.987-1.033) | 0.455 |
| DNT | 0.999 (0.991-1.007) | 0.890 | 0.998 (0.981-1.012) | 0.860 |
| NIHSS at admission | 1.127 (1.094-1.162) | **<0.001** | 1.165 (1.115-1.218) | **<0.001** |
| TOAST classification | 0.867 (0.742-0.867) | 0.123 | 0.558 (0.371-0.795) | **0.011** |
| OTT | 1.000 (0.999-1.002) | 0.368 | 1.001 (0.999-1.003) | 0.223 |
| Prior antiplatelet/anticoagulant use | 0.890 (0.566-1.360) | 0.661 | 0.990 (0.394-2.185) | 0.984 |
| **Laboratory signs** | | | | |
| WBC | 1.188 (1.113-1.268) | **<0.001** | 1.309 (1.174-1.456) | **<0.001** |
| Glucose | 0.993 (0.962-1.002) | 0.472 | 1.151 (1.022-1.271) | **0.030** |
| AST | 1.028 (1.015-1.042) | **<0.001** | 1.028 (1.006-1.046) | **0.017** |
| ALT | 0.978 (0.957-0.996) | 0.061 | 0.962 (0.911-1.002) | 0.187 |
| GGT | 0.998 (0.992-1.002) | 0.576 | 0.991 (0.971-1.003) | 0.347 |
| TC | 1.076 (0.974-1.183) | 0.214 | 1.053 (0.851-1.268) | 0.670 |
| Albumin | 0.989 (0.945-1.034) | 0.677 | 1.017 (0.930-1.108) | 0.755 |
| AKP | 1.003 (0.996-1.010) | 0.412 | 1.008 (0.994-1.020) | 0.326 |
| PTA | 0.964 (0.950-0.978) | **<0.001** | 0.963 (0.940-0.987) | **0.008** |
| Platelet | 0.994 (0.991-0.997) | **0.001** | 0.985 (0.978-0.992) | **0.001** |
| **Fibrosis scores** | | | | |
| FIB-4 | 1.585 (1.414-1.785) | **<0.001** | 1.590 (1.355-1.880) | **<0.001** |
| mFIB-4 | 1.151 (1.107-1.199) | **<0.001** | 1.167 (1.103-1.237) | **<0.001** |
| FIB-5 | 0.914 (0.886-0.943) | **<0.001** | 0.875 (0.827-0.925) | **<0.001** |
| APRI | 3.423 (1.867-6.706) | **<0.001** | 2.985 (1.379-6.183) | **0.011** |
| FORNS | 1.243 (1.118-1.385) | **<0.001** | 1.726 (1.332-2.254) | **<0.001** |
| ARR | 1.936 (1.520-2.467) | **<0.001** | 2.262 (1.511-3.276) | **<0.001** |
| AARPRI | 2.007 (1.621-2.500) | **<0.001** | 2.367 (1.711-3.275) | **<0.001** |
| FibroQ | 1.146 (1.106-1.190) | **<0.001** | 1.157 (1.098-1.221) | **<0.001** |
| Fibrosis index | 2.631 (1.922-3.638) | **<0.001** | 2.916 (1.653-5.116) | **0.002** |

**Abbreviations**: OR, odds ratio; CI, confidence interval; HT, hemorrhagic transformation; NIHSS, national Institutes of Health Stroke Scale; CHD, coronary heart disease; SBP, systolic blood pressure; DBP, diastolic blood pressure; mRS, modiﬁed Rankin Scale; DNT, door to Needle Time; TOAST, Trial of ORG 10172 in Acute Stroke Treatment; OTT, onset to treatment time; WBC, white blood cell; AST, aspartate aminotransferase; ALT, alanine aminotransferase; GGT, gamma-glutamyl transpeptidase; TC, total cholesterol; AKP, alkline phosphatase; PLT, platelet count; LDL-C, low-density lipoprotein cholesterol; HDL-C, High-density lipoprotein cholesterol; INR, international normalized ratio.
