# Supplementary figures and images for: The clinical value of fibrosis indices for predicting the hemorrhagic transformation in patients with acute ischemic stroke after intravenous thrombolysis
Source: Front Aging Neurosci. 2024 Nov 25;16:1492410. doi: 10.3389/fnagi.2024.1492410 (PMC11625795; doi:10.3389/fnagi.2024.1492410)

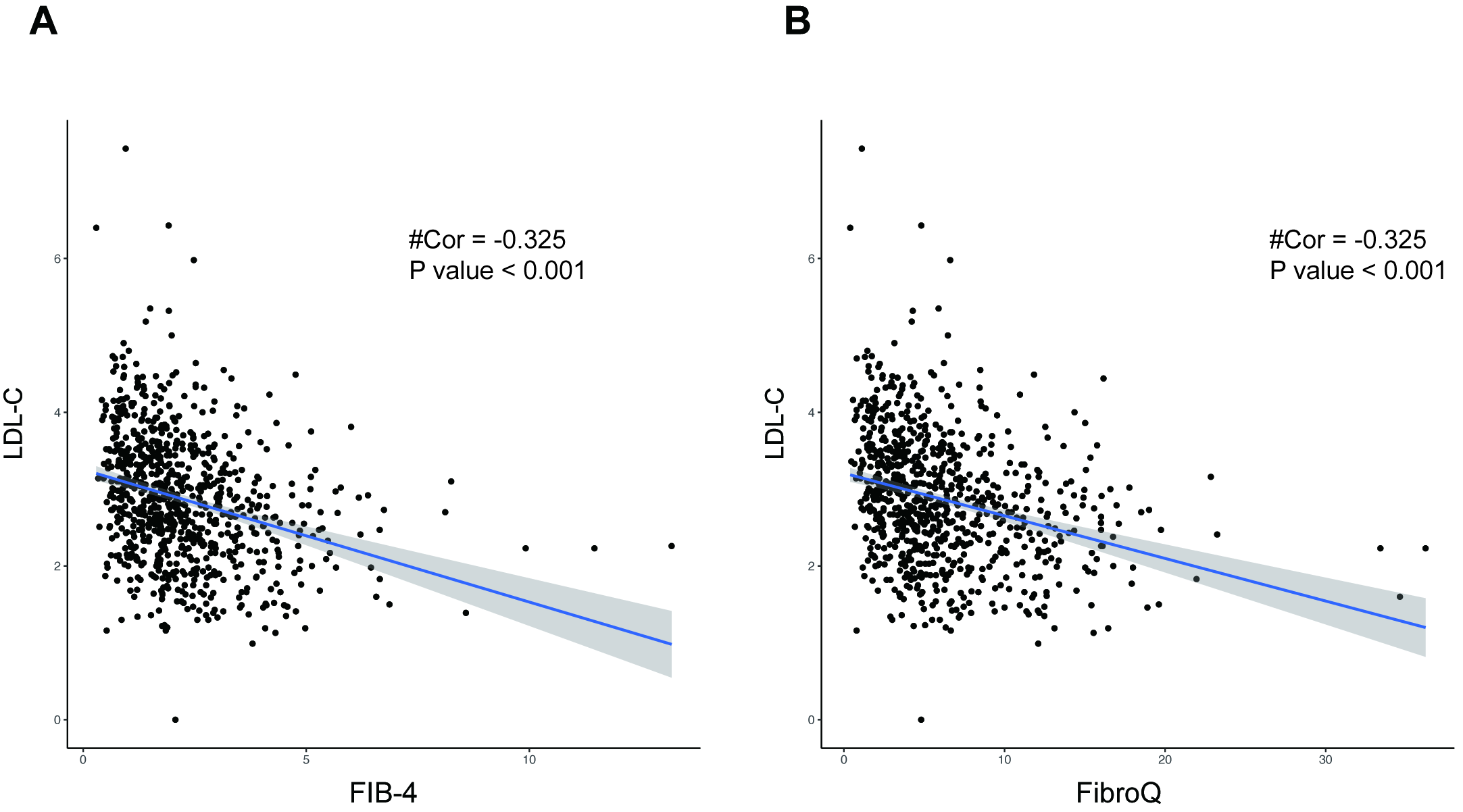

Supplement: Supplementary Figure 1 — The spearman’s rank correlation between two fibrosis indices and LDL- C. LDL-C, low-density lipoprotein cholesterol. [file Image_1.tif]
